# Multiple Sclerosis-Associated hnRNPA1 Mutations Alter hnRNPA1 Dynamics and Influence Stress Granule Formation

**DOI:** 10.3390/ijms22062909

**Published:** 2021-03-12

**Authors:** Joseph-Patrick W. E. Clarke, Patricia A. Thibault, Hannah E. Salapa, David E. Kim, Catherine Hutchinson, Michael C. Levin

**Affiliations:** 1 Department of Health Sciences, College of Medicine, University of Saskatchewan, Saskatoon, SK S7N 5E5, Canada; 2 Office of the Saskatchewan Multiple Sclerosis Clinical Research Chair, University of Saskatchewan, Saskatoon, SK S7K 0M7, Canada; patricia.thibault@usask.ca (P.A.T.); hes763@mail.usask.ca (H.E.S.); c.hutchinson@usask.ca (C.H.); 3 Department of Medicine, Neurology Division, University of Saskatchewan, Saskatoon, SK S7N 0X8, Canada; 4 Department of Anatomy, Physiology and Pharmacology, University of Saskatchewan, Saskatoon, SK S7N 5E5, Canada; david.kim@usask.ca

**Keywords:** hnRNPA1, mutations, optogenetics, stress granules, multiple sclerosis

## Abstract

Evidence indicates that dysfunctional heterogeneous ribonucleoprotein A1 (hnRNPA1; A1) contributes to the pathogenesis of neurodegeneration in multiple sclerosis. Understanding molecular mechanisms of neurodegeneration in multiple sclerosis may result in novel therapies that attenuate neurodegeneration, thereby improving the lives of MS patients with multiple sclerosis. Using an in vitro, blue light induced, optogenetic protein expression system containing the optogene Cryptochrome 2 and a fluorescent mCherry reporter, we examined the effects of multiple sclerosis-associated somatic A1 mutations (P275S and F281L) in A1 localization, cluster kinetics and stress granule formation in real-time. We show that A1 mutations caused cytoplasmic mislocalization, and significantly altered the kinetics of A1 cluster formation/dissociation, and the quantity and size of clusters. A1 mutations also caused stress granule formation to occur more quickly and frequently in response to blue light stimulation. This study establishes a live cell optogenetics imaging system to probe localization and association characteristics of A1. It also demonstrates that somatic mutations in A1 alter its function and promote stress granule formation, which supports the hypothesis that A1 dysfunction may exacerbate neurodegeneration in multiple sclerosis.

## 1. Introduction

Multiple sclerosis (MS) is an autoimmune disease with a significant neurodegenerative component characterized by inflammation-mediated demyelination of neuronal axons, and subsequent neurodegenerative axonal and neuronal cell loss [1]. Current research posits a multitude of molecular mechanisms underlying MS-associated neurodegeneration, many of which likely interact to initiate and exacerbate each other. Studies have shown that mitochondrial injury, microglial activation, reactive oxygen species, and apoptosis contribute to axonal injury [2,3,4,5,6,7,8]. Other studies implicate increased energy demands and the reduction of ATP production in axons, resulting in ‘virtual hypoxia’ [9,10,11]. Neurodegeneration can also be driven by inflammatory cytokines, autoantibodies, a lack of trophic support from myelin, glutamate toxicity, endoplasmic stress, altered iron homeostasis, and abnormal sodium channel expression on, and calcium accumulation within, damaged axons [12,13,14,15,16,17,18,19,20,21,22]. In addition, our lab and others have demonstrated a substantial role for RNA binding protein (RBP) dysfunction, including that of heterogeneous nuclear ribonucleoprotein A1 (hnRNPA1, or A), in the pathogenesis of MS and relevant MS models [9,23,24,25,26,27,28,29,30,31].

**Figure 1:**
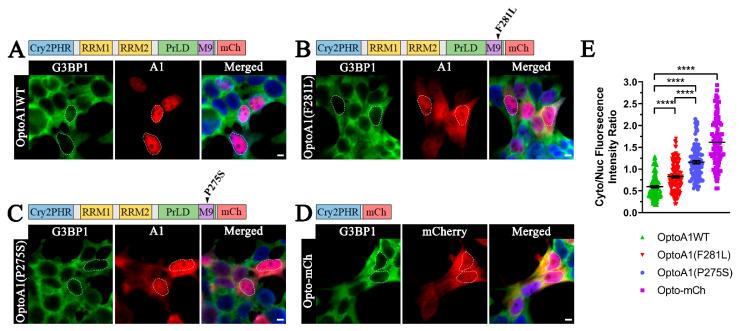
MS-associated A1 mutations F281L and P275S are mislocalized to the cytoplasm. Immunofluorescence of non-stimulated, HEK293Tcells expressing optogenetic fusion proteins of (**A**) full-length, wild-type A1 (OptoA1WT), (**B**) A1(F281L) (OptoA1(F281L)), (**C**) A1(P275S) (OptoA1(P275S)) and (**D**) Opto-mCh 12 h post-transfection. The cytoplasm is delineated by staining for G3BP1 (green), while nuclei are outlined (white outlines) using DAPI staining in merged images. Scale bars = 5 µm. (**E**) Cytoplasmic/nuclear fluorescence intensity ratio analysis of OptoA1 fusion proteins and Opto-mCh. The experimental ratios of five fields of view, per three independent experiments, were quantified and graphed in jitter plots, demonstrating that no one experiment drove the variation. Data shown are mean ± S.E.M. for three biological replicates. One-way ANOVA, with a Tukey post hoc test, was performed to indicate significance. **** *p* < 0.0001.

RBPs, including TAR DNA-binding protein 43 (TDP-43), Fused in Sarcoma (FUS) and T-cell-restricted intracellular antigen-1 (TIA1), and A1, function in many facets of RNA metabolism, as well as in other important cellular mechanisms [32,33,34]. For example, A1 plays a significant role in RNA splicing and RNA nucleocytoplasmic transport to ribosomal targets [35,36]. With two N-terminal RNA recognition motifs (RRMs) that bind RNA, it facilitates mRNA transit from nucleus to cytoplasm via the M9 nucleocytoplasmic transport domain found within its C-terminal prion-like domain (PrLD) (Figure 1A, schematic) [37,38,39]. The PrLD of A1 functions similarly to that of other RBPs and can mediate self- and non-self protein-protein interactions [40,41,42,43,44]. Reports from our lab have proposed that A1 self-interaction, mediated by M9 within the PrLD of A1, may lead to A1 protein dysfunction, and play a role in MS pathogenesis [25]. Specifically, our lab has previously shown the cytoplasmic mislocalization of A1 within neurons in the brains of MS patients, as well as in neurons from an MS-like animal model, experimental autoimmune encephalomyelitis (EAE) [28,29,30,31]. To molecularly understand how A1 becomes mislocalized and aggregated, our lab also assessed the mutation profile of A1 in cells from MS patients, and found an accumulation of significant somatic single nucleotide variants (SNVs) in the PrLD of A1 [25]. Further characterization showed that A1 PrLD mutations in the M9 sequence induce its mislocalization by prohibiting Transportin 1 (TNPO1) from properly binding to the M9 region and transporting A1 back to the nucleus [25,45,46]. This data is corroborated by studies in other neurodegenerative disorders (e.g., amyotrophic lateral sclerosis (ALS), frontotemporal lobar degeneration, and Alzheimer′s and Huntington′s diseases), showing that mutations within the PrLD of RBPs alter protein-protein interactions, leading to mislocalization and aggregate formation that influence cellular processes [47,48,49,50].

RBP mislocalization, aggregation, and dysfunction can be linked to altered cellular survival or death through alterations of stress granule (SG) dynamics [51,52,53]. SGs are membraneless organelles that form upon cell stress induction, and primarily function to protect mRNA and proteins from cell stress-associated degradative processes [54,55]. Upon the resolution of the stress, SGs dissociate and release their protected materials. Current research suggests that neurodegenerative disease-related RBP aggregation may affect SG formation and dissociation kinetics, but the mechanisms of how this may occur are still under debate. We and others hypothesize that protein aggregates may induce cell stress through the integrated stress response pathway or may form SGs under liquid-liquid phase separation mechanisms [43,51,53,56].

Current techniques to study and link protein aggregation dysfunction with altered cellular mechanisms in live cells and animals are limited. However, breakthroughs in protein optogenetics are showing promise in the field. One optogenetic approach uses the Cryptochrome 2 protein from *Arabidopsis thaliana*, which contains a photolyase-homologous region (Cry2PHR) that undergoes reversible homo-oligomerization in response to blue light (BL) [57]. Utilizing this protein, ALS researchers have recently shown spatiotemporal induction of TDP-43 aggregation in human induced pluripotent stem cell-derived neurons upon BL stimulation. Further, the authors showed that inhibiting TDP-43 aggregation by blocking self-interaction in the presence of aggregation light stimuli promotes neuron survival [50]. Another study has recently utilized this technique to spatiotemporally assemble SG-like membraneless organelles, without inducing a cell stress, and found that persistent or repetitive formation of SG-like assemblies led to cytotoxicity in U2OS cancer cells [58]. Thus, by coupling Cry2PHR optogenetics with our research in A1, we aimed to further understand A1 self-association dynamics in live cells and test whether altered A1 self-association dynamics affect SG induction without the treatment of an exogenous stressor.

In this study, we established an optogenetic system to permit finely tuned manipulation and kinetic analysis of A1 self-association and dissociation dynamics, and protein mislocalization. We demonstrate its utility by defining the self-association and dissociation dynamics, and cluster characteristics of two previously published MS-associated A1 mutants, F281L and P275S, located within the A1 M9 nucleocytoplasmic transport sequence of the PrLD [25]. This study shows that our A1 optogenetic system is representative of meaningful biological activity in the cell by connecting distinct A1 self-association clustering phenotypes to differences in the cells′ SG response. We demonstrate that A1 M9 mutations alter A1 self-association and dissociation cluster kinetics, and the quantity and size of A1 clusters within the cytoplasm of cells. We further show that A1 mutant clusters affect SG formation by increasing the occurrence of and A1 co-localization with SGs, as measured by the SG core protein Ras GTPase-activating protein-binding protein 1 (G3BP1). Together, these experiments provide a proof-of-concept for an A1 optogenetic system in assessing the molecular consequences of MS-associated A1 dysfunction in MS pathogenesis.

## 2. Results

### 2.1. A1 PrLD Mutations Exacerbate A1 Cytoplasmic Mislocalization

We first sought to expand on our original characterization of MS-associated somatic A1 mutant mislocalization by defining the individual influence of each A1 mutation on its nucleocytoplasmic mislocalization, altered protein-protein interactions, and attenuated cellular function [28,29,30,31]. We leveraged the optogenetic Cry2PHR protein tag to stimulate mutant and wild-type A1 self-association, coupled with live-cell imaging of A1 localization and multimerization dynamics. We first verified that wild-type and mutant A1 localization patterns were not affected by the Cry2PHR and mCherry tags, which were located on the N- and C- terminal of A1, respectively (see Cloning Methods). We evaluated the mislocalization of two previously described A1 PrLD mutants, F281L and P275S, in the absence of exogenous cellular treatments [25]. As most published MS mutations are present in progressive forms of MS, we chose two individual M9 mutations (F281L and P275S) that our lab previously discovered in patients with primary progressive MS [25]. Additionally, both mutations were previously discovered in the M9 region of the A1 PrLD, where TNPO1 binds, and while both clearly result in A1 cytoplasmic accumulation, the severity of this mislocalization has not been fully described [25]. To address this, we generated optogenetic Cry2PHR-A1-mCherry expression constructs of wild-type (WT; OptoA1WT) and mutant A1 (OptoA1(F281L) and OptoA1(P275S)) and expressed these in HEK293T cells for 12 h before subsequent experimentation and analysis (Figure 1A–C). As a negative control, we also designed and examined an empty Opto-mCherry (Opto-mCh) construct (Figure 1D). In the absence of BL stimulation, and with care taken to prevent exposure to environmental BL, we observed a predominant cytoplasmic fluorescence signal for the negative control Opto-mCh (Figure 1D,E), while the OptoA1WT (Figure 1A,E) demonstrated a predominant nuclear localization consistent with homeostatic A1 behavior. Consequently, when we compared WT and mutant A1 cellular expression in HEK293T cells via immunocytochemistry (ICC), we found that OptoA1 (F281L) and OptoA1 (P275S) both exhibited significantly increased cytoplasmic mislocalization as compared to OptoA1WT (Figure 1B,C,E), confirming previous observations by Lee et al. (2014) [25]. Interestingly, when the cytoplasmic expression of both mutants was quantified relative to nuclear signal, OptoA1 (P275S) mislocalized significantly more than OptoA1(F281L) (Figure 1E), expanding upon our previously published observations [25].

### 2.2. Blue Light Stimulation Facilitates Reversible A1 Self-Association without Inducing Cell Stress

**Figure 2:**
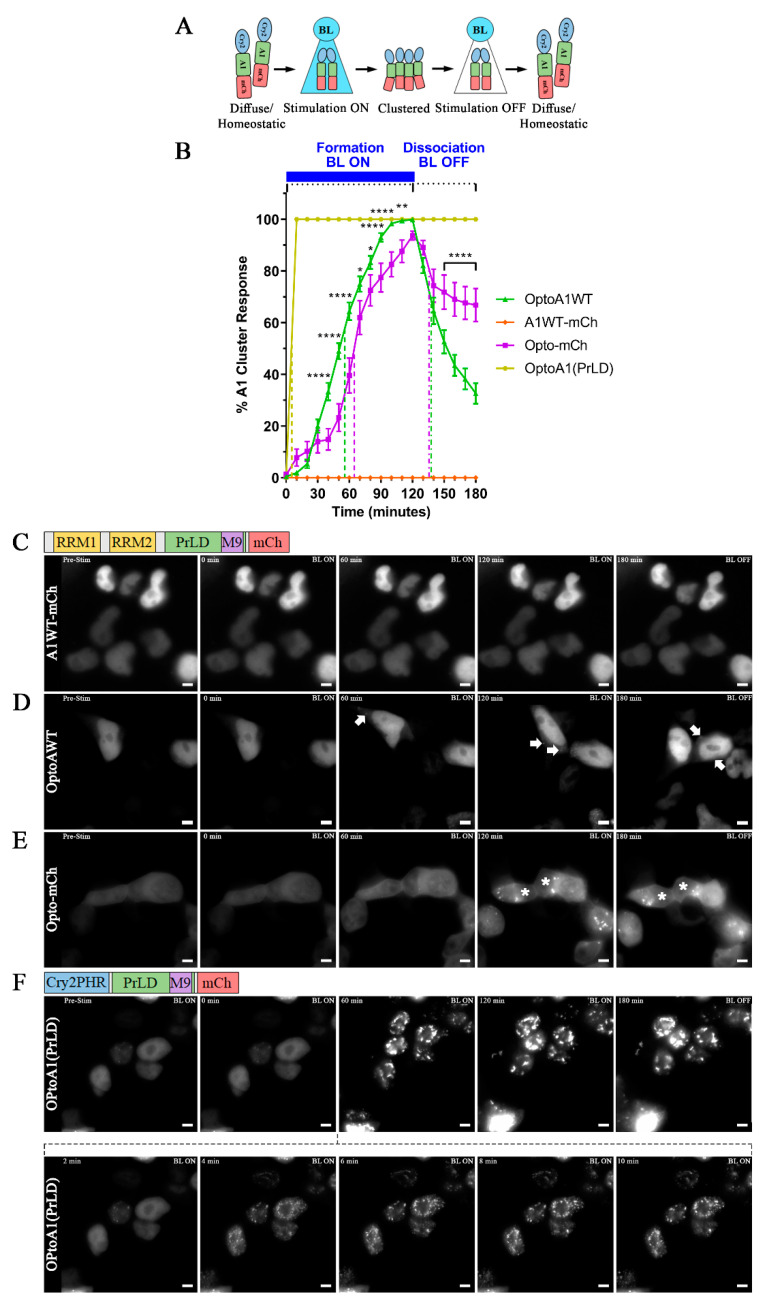
OptoA1WT responds differently to BL stimulation as compared to A1WT-mCh, Opto-mCh and OptoA1(PrLD). (**A**) Schematic of BL-inducible A1 self-association using the Cry2PHR optogene (blue), A1 protein (green) and mCh fluorescent tag (red). (**B**) Quantification of A1 cluster formation and dissociation during and following a BL stimulation protocol. HEK293T cells expressing OptoA1WT (green), A1WT-mCh (orange), Opto-mCh (magenta), or OptoA1(PrLD) (yellow) were exposed to BL stimulation (10,000 lux, 465 nm) for 120 min followed by 60 min of non-stimulation. Results are plotted as a percent maximum to the highest cluster response at 120 min for each OptoA1 construct, resulting in a kinetics curve for association and dissociation dynamics. Dashed lines indicate KA_1/2Max_, while dash-dotted lines indicate KD_1/2Max_. Data shown are mean ± S.E.M. for three biological replicates. Representative images of BL stimulated cells transfected with A1WT-mCh (**C**), OptoA1WT (**D**), Opto-mCh (**E**) and Opto-A1(PrLD) (**F**) are shown. Since Opto-A1(PrLD) forms clusters within minutes of BL exposure, both a long and short BL stimulation timeframe is shown in (**F**). Arrows indicate the formation of OptoA1 clusters. Stars indicate cells with Opto-mCh cluster (non-A1) formation. Scale bars = 5 µm. * *p* < 0.05; ** *p* < 0.01; **** *p* < 0.0001.

To establish the use of our OptoA1 system for kinetic analysis of A1 self-association and dissociation, we examined several controls to validate the reliability and accuracy of our system. First, we confirmed that the Opto-mCh tags themselves, and the transient protein expression of our system, do not drive protein clustering in the absence of BL stimulation. When we carried out live-cell imaging of Opto-transfected cells without BL stimulation over 180 min, there was no cluster formation by any of the OptoA1 constructs (WT, F281L, P275S) under the conditions we used for our subsequent experiments (200 ng plasmid DNA, beginning imaging at 12 h post-transfection) (Supplemental Figure S1A–C; Supplemental Video S1A–C). Next, we verified that BL stimulation specifically and exclusively drove clustering of Opto-mCh tagged proteins. Our BL stimulation paradigm (Figure 2A) consisted of BL exposure with 465 nm BL for 120 min (min; BL ON) to induce OptoA1 self-association, with quantification of cytoplasmic mCh A1 clusters every minute during stimulation. After 120 min, we ceased BL stimulation (BL OFF), and followed with 60 min of non-stimulation to allow for A1 cluster dissociation back into the protein’s monomeric form, again quantifying cytoplasmic mCh A1 clusters every minute (Figure 2A). Using this paradigm, we first verified that clustering is specifically facilitated by BL stimulation of the Opto-tagged protein by comparing the effects of BL stimulation of OptoA1WT and A1WT-mCh (lacking the Cry2PHR Opto-tag) on construct self-association and dissociation with live-cell imaging. We found OptoA1WT only responded to BL stimulation, while the A1WT-mCherry construct showed no cluster formation, confirming that A1 clustering was specifically facilitated by the Cry2PHR in response to BL stimulation (Figure 2B–D; Supplemental Video S2A,B).

To further attest that the observed effects of BL stimulation on OptoA1WT kinetics are defined by the properties of A1, and not due to the Opto and mCherry tags, we compared the effect of BL stimulation kinetics between OptoA1WT and Opto-mCh (lacking the A1 gene) (Figure 2B,E). We found that OptoA1WT association kinetics are distinct from Opto-mCh, where OptoA1WT formed cytoplasmic clusters at a half-maximal kinetic association rate (KA_1/2Max_) of 55 min, compared to a KA_1/2Max_ of 64 min for Opto-mCh (Figure 2B,D,E; Supplemental Video S2B,C). We also found that OptoA1WT clusters dissociated more efficiently than Opto-mCh clusters (67 ± 8% (S.E.M.; 95% confidence level (CL)) total dissociation from the max percent cellular cluster formation at the height of the stimulation cycle, compared to 33 ± 8% (S.E.M.; 95% CL), respectively], when comparing the percent maximal cluster response at 120 min to the end-point cluster response at 180 min (Figure 2B). These observations demonstrated that Opto-mCh kinetics are different than with inclusion of the A1 gene, which is consistent with other published reports on Opto-mCh [50,58,59], and that the properties of the A1 protein affect the self-association interaction dynamics of OptoA1.

We further described the specificity of A1 in our optogenetics system by BL stimulation of an Opto-PrLD construct that contained the entire PrLD of A1 without its RRMs (i.e., amino acids 191–320) and compared its kinetics with both full-length OptoA1WT and Opto-mCh (Figure 2B,D,F). Our results demonstrated that Opto-PrLD self-association kinetics were significantly faster than OptoA1WT and Opto-mCh [KA_1/2Max_ of 5 min], and that once formed, they did not dissociate upon cessation of BL, indicating that the PrLD of A1 drives the self-association effect, and that the Opto tag only facilitates in initiating self-interaction (Figure 2B,F; Supplemental Video S2D,E). These observations are strengthened by a previous report by Shin et al., wherein the authors performed similar experimentation with a Cry2-PrLD construct and found that the PrLD of A1 drives A1 self-association kinetics [59].

### 2.3. A1 Protein Self-Association Kinetics Are Altered by PrLD Mutations

**Figure 3:**
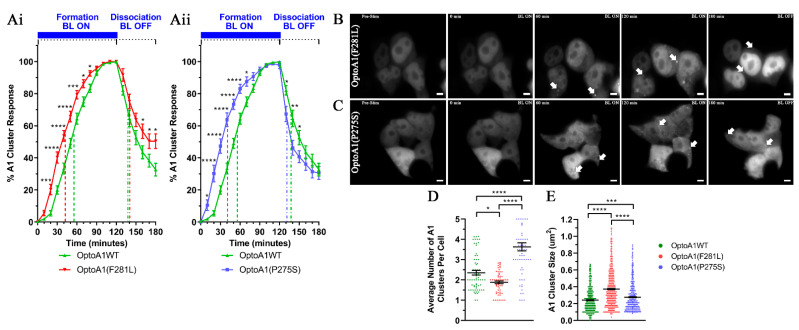
OptoA1(F281L) and OptoA1(P275S) mutants affect A1 self-association dynamics and characteristics. (**Ai**) and (**Aii**) Quantification of mutant A1 cluster formation and dissociation during and following a BL stimulation protocol. HEK293T cells expressing OptoA1WT (**Ai**, **Aii**; similar data used from Figure 2B), OptoA1(F281L) (**Ai**), or OptoA1(P275S) (**Aii**) were exposed to BL stimulation (10,000 lux, 465 nm) for 120 min followed by 60 min of non-stimulation. Results are plotted as a percent maximum to the highest cluster response at 120 min for each OptoA1 construct, resulting in a kinetics curve for association and dissociation dynamics. Curves are the result of three independent experiments. Dashed lines indicate KA_1/2Max_, while dash-dotted lines indicate KD_1/2Max_. Two-way ANOVA, with a Bonferroni post hoc test, was performed to indicate significance on kinetics curves. (**B**) and (**C**) Representative images of BL stimulated OptoA1(F281L) and OptoA1(P275S) clusters, respectively. Arrows indicate the formation of OptoA1 clusters. Scale bars = 5 µm. (**D**) Quantification of BL stimulated OptoA1 clusters per cell at peak stimulation (120 min of BL ON) combined from three biological experiments. (**E**) Quantification of BL stimulated OptoA1 clusters sizes at peak stimulation (120 min of BL ON) combined from three biological experiments. One-way ANOVA, with a Tukey post hoc test, was performed to indicate significance between cluster number and sizes. Data shown are mean ± S.E.M. for three biological replicates. * *p* < 0.05; ** *p* < 0.01; *** *p* < 0.001; **** *p* < 0.0001.

Having established the utility of the OptoA1 system for examining the kinetics of A1 self-association, we then used this system to determine the effects that A1 mutations have on its association/dissociation dynamics. As noted above, with BL ON, OptoA1WT formed cytoplasmic clusters at a KA_1/2Max_ of 55 min, while after BL OFF, OptoA1WT clusters dissociated back into monomeric species at a KD_1/2Max_ of 18min (i.e., 18 min after BL stimulation has ceased at 120 min, or 138 min from the start of experimentation) (Figure 2B,D; Supplemental Video S2B). When we introduced the OptoA1(F281L) mutation, the associative kinetics of A1 occurred significantly faster with a KA_1/2Max_ of 42 min (Figure 3Ai,B; Supplemental Video S3A). The dissociative kinetics for OptoA1(F281L) occurred similarly to OptoA1WT, with a KD_1/2Max_ of 21 min; however, OptoA1WT clusters dissociated more efficiently than OptoA1(F281L) clusters, with 67 ± 8% (S.E.M.; 95% confidence level (CL)) total dissociation from the max percent cellular cluster formation at the height of the stimulation cycle, compared to F281L total dissociation of 49 ± 9% (S.E.M.; 95% CL) (Figure 3Ai; Supplemental Video S3A). OptoA1(P275S) also caused faster cluster formation, with a KA_1/2Max_ of 40 min (Figure 3Aii,C; Supplemental Video S3B), but surprisingly, also dissociated significantly faster than OptoA1WT clusters initially, with a KD_1/2Max_ of 11 min (Figure 3Aii; Supplemental Video S3B), while ultimately achieving a similar total dissociation to OptoA1WT (69 ± 8% total dissociation (S.E.M.; 95% CL)). Critically, and in comparison to Opto-mCh (Figure 2E) or OptoA1(PrLD) lacking RRMs (Figure 2F), we only observed OptoA1 cluster formation for all three full-length A1 constructs in the cytoplasm, with nuclear OptoA1 being predominantly diffuse, regardless of mutation (Figure 2D and Figure 3A,B; Supplemental Videos S2B and S3A,B). This further demonstrates that cluster formation is regulated by and representative of characteristics of the A1 protein itself. Thus, we find that A1 PrLD mutations affect A1 clustering kinetics and show that each mutation has a distinct impact.

### 2.4. A1 Protein Cluster Characteristics Are Altered by PrLD Mutations

A1 clusters formed during the kinetics study were also analyzed based upon the average number of cytoplasmic OptoA1 clusters formed per cell over the course of the BL induction experiment. Overall, we observed significantly fewer clusters per cell with OptoA1(F281L) and significantly more OptoA1(P275S) clusters per cell as compared to OptoA1WT cells (Figure 3D). In addition, we found that the average cluster sizes of OptoA1(F281L) and OptoA1(P275S) were both significantly larger than OptoA1WT clusters (Figure 2E). Interestingly, the cluster size difference between OptoA1(F281L) and OptoA1(P275S) was also different, with OptoA1(F281L) forming significantly larger clusters (Figure 3E). This data indicates that the MS-associated A1 mutations not only influenced A1 self-association and dissociation dynamics, but also affected the physical characteristics of A1 clusters.

### 2.5. A1 Protein Clusters Co-Localize with Stress Granules

Having established the specificity and dynamics of OptoA1 self-association, we next sought to confirm that OptoA1 self-association has biologically relevant effects on the cell. Considering that the literature has established SG formation in response to A1 mislocalization in multiple systems [26,27,30], we sought to monitor SG formation kinetics in response to OptoA1 cluster formation. Initially, we verified that BL alone does not induce a cellular stress and confound our system by stimulating non-transfected HEK293T cells for 120 min, followed by Western immunoblotting for phosphorylated elongation initiation factor 2α (p-eIF2α). eIF2α is non-phosphorylated under homeostatic conditions, but is rapidly phosphorylated during cell stress, which induces the ISR pathway. As a positive control for cell stress, we treated non-transfected HEK293T cells with sodium arsenite (NaAsO_2_) for 30 min, and as a negative control, collected protein from non-transfected, non-treated HEK293T cells. Our results show that BL stimulation alone did not induce cell stress in our HEK293T cells, as indicated by a lack of p-eIF2α signal in our BL treated samples, and thus would not be a variable in our subsequent kinetic studies (Supplemental Figure S2).

**Figure 4:**
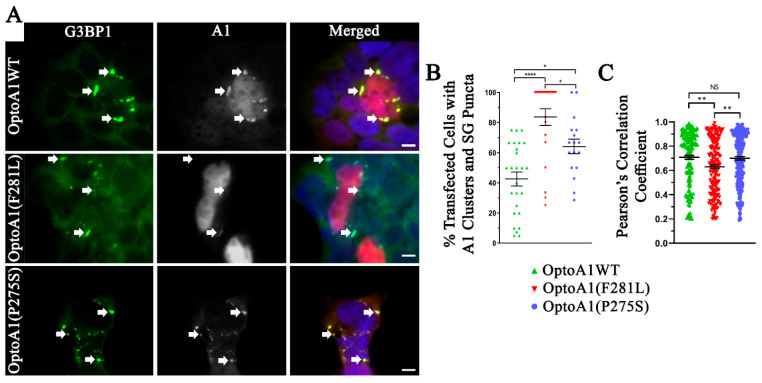
Cells containing OptoA1(F281L) and OptoA1(P275S) mutant clusters have more SG formation and altered A1 to SG co-localization. (**A**) Immunofluorescence of 120-min BL stimulated HEK293T cells expressing OptoA1WT, OptoA1(F281L) and OptoA1(P275S) constructs (grey), co-staining for endogenous G3BP1-positive SGs (green). DAPI (blue) indicates the nuclei of cells in merged images. Arrows indicate OptoA1 cluster and SG puncta co-localization. Scale bars = 5 µm. (**B**) Quantification of the percent of transfected OptoA1 cells that contained both A1 clusters and G3BP1-positive SG. (**C**) Quantification of A1 cluster and G3BP1-positive SG co-localization. Data shown are mean ± S.E.M. for three biological replicates. Co-localization was performed using ImageJ Coloc2 assessed using Pearson’s correlation coefficients. One-way ANOVA, with a Tukey post hoc test, was performed to indicate significance between percent transfected cells with A1 clusters and SG puncta, and Pearson′s correlation coefficients. * *p* < 0.05; ** *p* < 0.01; **** *p* < 0.0001; NS = Not Significant.

We next examined OptoA1WT transfected HEK293T cells after 120min of BL stimulation for SG formation. When we stained the cells for endogenous G3BP1, the core SG marker protein, these cells showed a SG-like response comparable to NaAsO_2_ treatment (positive control for SG formation) (Figure 4A; Supplemental Figure S3) [60]. To further assess whether we formed endogenous SGs, or non-specific SG-like structures, we co-stained for TDP-43, a protein shown in literature to co-localize with SGs upon their formation, in both BL-stimulated and NaAsO_2_-treated cells [61]. We observed co-localization between G3BP1, TDP-43 and OptoA1WT in our SG structures, indicating that OptoA1WT clusters can promote endogenous SG formation (Figure 4A; Supplemental Figure S3).

When we analyzed OptoA1(F281L) and OptoA1(P275S), we found a significant increase in the formation of SGs as indicated by G3BP1 punctum formation, as compared to OptoA1WT (Figure 4A,B). Interestingly, OptoA1(F281L)-transfected cells further showed significantly more SG formation than OptoA1(P275S) (Figure 4A,B). In cells with both A1 clusters and SGs, we analyzed co-localization between OptoA1 and G3BP1-positive SGs. OptoA1WT and OptoA1(P275S) both showed a very strong positive correlation with SG localization with no significant difference; while OptoA1(F281L) clusters also showed a strong, positive correlation with SG localization, it was significantly lower than with the other two constructs (Figure 4C). Overall, this data indicates that mutations in A1 can influence the formation of SGs.

### 2.6. A1 Protein Clustering Promotes Stress Granule Formation

**Figure 5:**
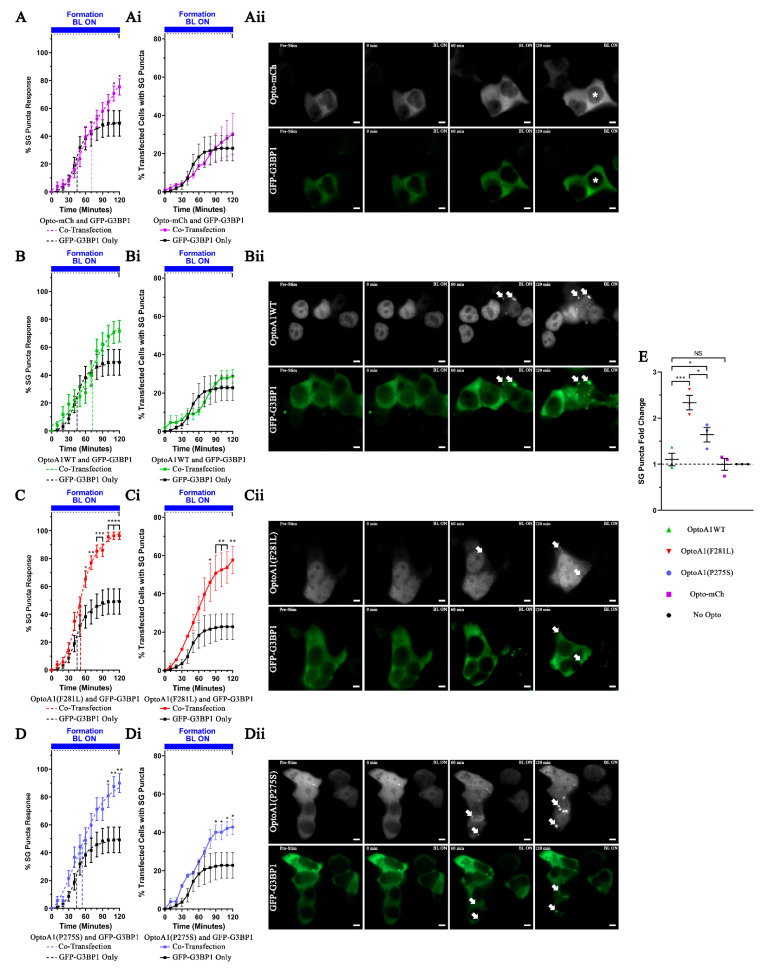
SG formation dynamics are increased in cells expressing OptoA1(F281L) and OptoA1(P275S) mutants. Quantification of SG puncta formation and dissociation during a BL stimulation protocol. HEK293T cells expressing GFP-G3BP1 alone (**A**–**D**; black curves), or co-expressing Opto-mCh (**A**; magenta), OptoA1WT (**B**; green), OptoA1(F281L) (**C**; red), or OptoA1(P275S) (**D**; blue) with GFP-G3BP1 were exposed to BL stimulation (10,000 lux, 465 nm) for 120 min. Results are plotted as a percent maximum to the highest SG puncta response at 120 min for each OptoA1 construct co-transfected with GFP-G3BP1, resulting in a kinetics curve for association dynamics. Dashed lines indicate KA_1/2Max_. Two-way ANOVA, with a Bonferroni post hoc test, was performed to indicate significance on kinetics curves. (**Ai**), (**Bi**), (**Ci**) and (**Di**) Quantification of percent cells with SG puncta over the time-course of a BL experimental paradigm. Two-way ANOVA, with a Bonferroni post hoc test, was performed to indicate significance on kinetics curves. (**Aii**), (**Bii**), (**Cii**) and (**Dii**) Representative images of 120-min BL stimulated, GFP-G3BP1 and Opto-mCh (**A**), OptoA1WT (**B**), OptoA1(F281L) (**C**), or OptoA1(P275S) (**D**) co-transfected cells. Arrows indicate OptoA1 cluster and SG puncta co-localization. * indicate cells with SG puncta formation without A1 co-localization. Scale bars = 5 µm. (**E**) Fold change quantification of SG puncta formation in cells with A1 clusters. One-way ANOVA, with a Tukey post hoc test, was performed to indicate significance between fold change SG puncta formation in OptoA1 cluster containing cells at peak stimulation (120 min). Data shown are mean ± S.E.M. for three biological replicates. * *p* < 0.05; ** *p* < 0.01; *** *p* < 0.001; **** *p* < 0.0001.

To determine whether the formation of A1 clusters directly affect SG formation, we next paralleled our exploration of cluster association kinetics in HEK293T cells transfected with both OptoA1 and a GFP-tagged G3BP1 protein. Previous literature has shown that G3BP1 over-expression forms SGs non-specifically [62,63,64], and so we determined that the low levels of SG formation caused by GFP-G3BP1 transfection alone (i.e., without OptoA1 co-transfection) (Figure 5A–D; black curve) represents baseline SG formation in our system. We considered G3BP1 SG formation varying from this baseline to be directly affected by OptoA1 cluster formation, since we determined that BL alone did not induce cell stress (Supplemental Figure S3). To compare the effects of OptoA1 on SG formation, we determined the baseline KA_1/2Max_ SG formation response to be 46 min, with a maximal SG puncta response of 49 ± 9% (S.E.M.; 95% CL) (Figure 5A–D; black line). In addition, we assessed the effect of the Opto and mCherry tags on SG formation using Opto-mCh (Figure 5A). We found that Opto-mCh cluster formation increased SG assembly KA_1/2Max_ by 24min above baseline, with a maximal SG puncta response of 75 ± 7% (S.E.M.; 95% CL) (Figure 5A; Supplemental Video S4A). We determined this effect represented a non-specific response to protein clustering, and as we observed with A1 kinetics, any alterations from this Opto-mCh standard would be because of A1. Interestingly, the proportion of SG-positive transfected cells formed by Opto-mCh did not significantly increase compared to GFP-G3BP1 only (Figure 5Ai,Aii,E), indicating that while Opto-mCh affected the rate of SG formation, it did not affect maximal SG formation.

When we analyzed the OptoA1 constructs, we found that OptoA1WT cluster formation caused SG assembly with a KA_1/2Max_ of 27 min above baseline (Figure 5B; Supplemental Video S4B), with a maximal SG puncta response of 72 ± 8% (S.E.M.; 95% CL) (Figure 5B); both analyses were not significantly different than Opto-mCh, indicating that OptoA1WT clustering did not have a significant effect on SG formation over non-specific protein clustering (Figure 5A,B,E). Further, the number of SG-positive transfected cells did not significantly increase compared to GFP-G3BP1 only (Figure 5Bi,Bii,E), nor did they significantly differ from Opto-mCh (Figure 5Ai,Aii,Bi,Bii,E). When we assessed OptoA1(F281L) and OptoA1(P275S), however, we observed SG formation occurred significantly earlier and with a higher maximal SG puncta response for both, with a KA_1/2Max_ 5min above baseline, 96 ± 3% maximal (S.E.M.; 95% CL) (F281L: Figure 5C; Supplemental Video S4C), and 9min above baseline, 90 ± 7% maximal (S.E.M.; 95% CL) (P275S: Figure 5D; Supplemental Video S4D). Additionally, we found significantly more transfected cells developed SGs for each mutant (Figure 5Ci,Cii,Di,Dii,E), echoing our single-timepoint endogenous SG imaging results observed above (Figure 4B). Further analysis of this data showed a significant 2.3- and 1.6-fold change increase in SG formation from baseline SG formation with OptoA1(F281L) and OptoA1(P275S) co-transfection, respectively (Figure 5E). Cumulatively, these data show that OptoA1 clustering specifically impacts SG formation kinetics, and more importantly, that clustering of OptoA1 mutants that have faster association kinetics also increases SG formation rates and total accumulation above OptoA1WT.

## 3. Discussion

Our lab previously demonstrated a link between A1 dysfunction and progressive forms of MS [9,23,24,25,26,27,28,29,30,31]. To help further understand A1 molecular mechanisms and establish methodology to study A1 mutant species in vitro and their effects on A1 dysfunction, we developed an optogenetic system that induces A1 mislocalization and aggregation and recapitulates pathological features of A1 observed in central nervous system (CNS) tissue from MS patients and animal models of disease [28,29,31]. Similar optogenetic systems have been used recently to study the dynamics of PrLDs in RBPs (e.g., FUS and A1) and how their biophysical properties influence self-interaction dynamics. In addition, studies have utilized optogenetics to focus upon other RBPs (e.g., TDP-43, G3BP1) whose self-association properties are associated with neurodegenerative diseases, but prior to this study none have been developed to study full-length wild-type A1, nor to describe the effects of MS-associated A1 mutations [50,58,59,65].

Using our optogenetic system, we demonstrate that cytoplasmic mislocalization of A1 is mediated by MS-associated mutations F281L and P275S in the M9 nucleocytoplasmic transport sequence, itself contained within A1’s PrLD, and that the addition of an optogenetic and fluorescent protein tag does not disrupt this phenotype (Figure 1). We then defined the specificity of a BL stimulation paradigm with the optogenetic protein tag (Figure 2; Supplemental Figure S1; Supplemental Video S1A–C; Supplemental Video S2A–E) and used this to confirm other published findings that the A1 PrLD also rapidly forms nuclear clusters upon BL stimulation in our system (Figure 2B,F) [59]. We then demonstrated that the MS-linked A1 mutations F281L and P275S also promote A1 to self-associate more readily when induced with a BL stimulus, and further found that these mutants show altered dissociation properties compared to OptoA1WT when stimulation ceased (Figure 3; Supplemental Video S3A,B). Interestingly, when we compared the interaction dynamics of OptoA1WT and Opto-mCh, we also observed a variance in effects, whereby OptoA1WT dissociated more efficiently than Opto-mCh (Figure 2; Supplemental Video S2B,C). We hypothesize that the discrepancy in dissociation can be attributed to endogenous mechanisms that target and resolve protein self-interaction clustering (e.g., heat-shock molecular chaperone proteins, degradation via the proteasome, and/or autophagy); the OptoA1WT construct contains A1, an endogenous full-length protein with endogenous responsiveness to such mechanisms, whereas Opto-mCh is an entirely foreign protein not adapted to mammalian protein regulation pathways. As another endogenous mechanism of A1 cluster clearance, TNPO1 may aid in dissociation by shuttling resolved monomeric OptoA1WT back to the nucleus, thereby reducing the local cytoplasmic concentration of OptoA1WT and limiting further self-interactions. This proposed mechanism is supported by a recent study by Hutten et al. showing that increasing concentrations of importin proteins inhibits TDP-43 cytoplasmic self-association by transporting it back into the nucleus [66].

Fixed-imaging studies of multisystem proteinopathy (MSP) and ALS have also found that A1 mutations affect its self-association, suggesting a common mechanism of dysregulation between diseases with associated A1 mutations [42,43,67]. Our observations are the first to use a live-cell system to describe how MS-associated A1-M9 PrLD mutations have a distinct effect on self-association dynamics and have on A1 dysfunction and its downstream cellular processes. We assessed the influence of mutations in the M9 sequence on A1 self-association, as our lab previously showed that M9 mutations in A1 alter TNPO1 binding and influence cytoplasmic mislocalization. Considering that some patients with progressive MS had multiple mutations in the TPNO1 binding domain of A1 [25], future studies will assess the cumulative impact of multiple mutations on A1 aggregation dynamics and its relationship to the pathogenesis of MS.

Previous research has shown that A1-M9 mutations attenuate the ability of TNPO1 to bind to the M9 sequence, and in turn, decrease the capacity of A1 to re-localize back to the nucleus after delivering its RNA cargoes to cytoplasmic ribosomes for translation [25]. This results in increased cytoplasmic concentrations of A1, and we hypothesize that this accumulation facilitates promiscuous self-interactions that can lead to proteinopathic cluster formation. However, the influence of missense somatic mutations in the M9 region of the A1 PrLD on protein trafficking and self-association is not completely understood; these mutations may directly change the A1:A1 or A1:TNPO1 interfaces, or they may introduce broader structural changes that also impact post-translational modifications or A1:protein interactions. Both mechanisms are supported by data from previous studies in MSP and ALS, where it has been shown that disease-associated mutations generate potent steric zippers in the PrLD of A1 and allowing A1 clusters to form more easily [42,43,67].

Whether or not a direct effect of local structural changes due to A1 mutations, we found that mutant A1 self-associated clusters are characteristically different from A1 wild-type clusters, based on size and numbers per cell (Figure 3D,E). Again, as we observed with mislocalization and kinetics, we found a dissimilarity in characteristics depending on mutation type, with A1(F281L) and A1(P275S) both forming larger clusters than A1WT, but A1(F281L) forming fewer and A1(P275) forming more clusters compared to A1WT. These results suggest that the local amino acid sequence in A1 affects its dynamics, but furthermore, demonstrate that cluster dynamics are not perfectly correlated with cluster size or number, indicating that these metrics define distinct mechanisms of protein clustering. More broadly, considering the previous observations that a multitude of somatic mutations can occur in the PrLD of A1, each mutation may have a significantly varying effect on A1 dysfunction and dysregulation [25,42,43,67].

Downstream of dysfunctional RNA binding, we further hypothesized that the alterations in A1 self-association kinetics altered SG formation. Previous evidence has shown that A1 and SGs co-localize in MS patient brain tissue, and that cells subjected to exogenous IFNγ showed A1 mislocalization, as well as formation of SGs that colocalized with the mislocalized A1 [26,27]. Based upon these observations, and utilizing our optogenetic system, we found that mutant A1 cluster formation itself specifically influenced the formation of SGs, although wild-type A1 did not (Figure 4 and Figure 5; Supplemental Video S4A–D). We hypothesize that mutant A1 cluster formation may influence SG formation in two different ways, and the relative contribution of each may be driven by the mutants’ distinct kinetics and characteristics of cluster formation. First, the formation and accumulation of A1 clusters themselves may induce cell stress, either by overtaxing protein degradation systems (e.g., proteasomal degradation, autophagy), or by activating molecular stress pathways (e.g., integrated stress response). In this model, gradual aberrant accumulation of A1 clusters induced by somatic mutations causes sustained cell stress, leading to continual SG formation and eventual cell death. This idea is partially corroborated by a study showing that HeLa cells depleted of A1 using RNAi were less viable and recovered poorly after exposure to an osmotic cell stress [68]. Although A1 cluster formation does not deplete total cellular A1, we propose that mislocalization causes molecular dysfunction similar to depletion. Second, we hypothesize that the formation of A1 clusters may indirectly induce liquid-liquid phase separation (LLPS) of PrLD-containing proteins, many of which are found in SGs (e.g., G3BP1, TDP-43, FUS and TIA1), thereby catalyzing SG assemblies independent of cell stress signaling [43,54,56,69,70]. SG formation has been shown to be driven by concentration-dependent A1 LLPS and requires an A1 protein concentration threshold to form [71], but may be enhanced specifically by clustered forms of A1 [58]. As neither study directly examined the influence of A1 cluster formation on cell stress, we currently propose that both mechanisms may contribute to SG formation in our system.

How these A1 mutations contribute to the pathogenesis of MS is not yet clear and requires further study. Herein, we show the molecular consequences of A1 mutations. These mutations may cause cellular dysfunction in either the effector (immune system) or target (CNS), both of which are relevant to the pathogenesis of MS. In terms of the immune response, A1 dysfunction has profound effects on the immune response [72,73,74] and other SNVs (e.g., hypoxanthine-guanine phosphoribosyl transferase (HPRT)) have been shown to alter the immunity of MS patients [75,76,77,78]. In the CNS, genetic mutations in A1 have been shown to contribute to the pathogenesis of ALS [42,79,80], which like MS, is a progressive disease with a significant neurodegenerative component [42,81,82]. Importantly, somatic mutations in both the immune and nervous system may co-exist (mosaicism) and cause neurologic disease [78,83,84,85]. Future studies, which we have initiated in our lab, will address the contribution of A1 dysfunction to the pathogenesis of MS in both the immune system and neurons of the CNS.

In summary, our data provide novel molecular insights into how MS-associated A1 mutations influence endogenous A1 function, and the downstream effects of A1 dysregulation in cells. This work adds to previous literature showing that A1 mislocalization and dysfunction may contribute to the pathogenesis of MS. Importantly, we have established a tunable and specific optogenetic A1 clustering system with the capacity for live imaging and kinetic analysis and demonstrated its utility in examining the effects of wild-type and mutant A1 clustering on cell biology. Although these studies are limited in that they did not specifically address how these mutations cause cell death, ongoing studies will address the effects of A1 mutations on markers of cell death and dysfunction in neurons.

## 4. Materials and Methods

### 4.1. Patient Material

The mutations used in this study were reported previously [25]. Of the two mutations that were studied, both were isolated from primary progressive MS patients [25]. The first mutation (P275S) was a 59 year-old, white male who had an expanded disability status scale (EDSS) of 6. The second was a 61 year-old, white male with an EDSS 6.5. There is no radiologic information on the MS patients.

### 4.2. Cell Culture

Human embryonic kidney 293T (HEK293T) cells (female, purchased from ATCC, Manassas, VA, USA) were maintained in DMEM (Thermo Fisher Scientific, Burlington, ON, Canada) supplemented with 10% fetal bovine growth serum (Thermo Fisher Scientific, Burlington, ON, Canada) and 1% penicillin/streptomycin (Thermo Fisher Scientific, Burlington, ON, Canada) at 37 °C and 5% CO_2_. Cells were seeded onto 4-well glass bottom slides (Fisher Scientific, Ottawa, ON, Canada) or 8-well slides (Fisher Scientific, Ottawa, ON, Canada) coated with poly-d-lysine (50 mg/mL) (Millipore Sigma, Oakville, ON, Canada), or 6-well plates (Fisher Scientific), and incubated for 24 h prior to transfections (Lipofectamine 2000, Thermo Fisher Scientific, Burlington, ON, Canada) with 200 ng (4- and 8-well slides) or 2.0 µg (6-well plate) of DNA. Cell cultures were lightly covered with aluminum foil after transfection to provide protection from ambient light exposure and non-specific activation of the optogenetic protein, and all manipulations were performed in ambient light-free conditions.

### 4.3. Cloning

Full-length (Gene ID: 3178; Accession #: NM_002136.4) and mutated forms of the A1 gene were previously isolated and cloned into pTriEx plasmids [25]. Using pTriEx-A1 plasmids, N-pmCry2PHR-A1-mCherry-C plasmids were constructed in a two-step process using Gibson Assembly cloning (HiFi DNA Assembly Master Mix, NEB). To note, we positioned the Cry2PHR on the N-terminus of A1 to distance it away from the A1 PrLD domain, so that it would not non-specifically influence A1 interaction dynamics, and to allow for the assessment of A1 PrLD mutations for their influence on the overall kinetics of self-interaction. Step one created A1 full-length and mutant genes tagged with the mCherry gene, while step two added the Cry2PHR gene. Initially, A1 and mCherry genes were cloned into the pmCry2PHR-mCherry (mCh) base vector (Plasmid 26866, Addgene, Watertown, MA, USA) between the NheI and MfeI restriction enzyme sites of the pmCry2PHR-mCh backbone, with the two-fragment Gibson Assembly designed to reconstruct the NheI restriction site. A short flexible amino acid linker of GGSG (ggaggatcagga) was inserted during Gibson Assembly between A1 and mCherry to separate both proteins and prohibit unwanted non-specific interactions. In step two, the Cry2PHR gene was cloned into the pmA1-mCherry base vector, created in step one, using two-fragment Gibson Assembly and the NheI restriction enzyme site. Again, a short GGSG (ggaggatcaggc) amino acid linker was inserted during Gibson Assembly between Cry2PHR and A1. The N-pmCry2PHR-A1(PrLD)-mCherry-C plasmid was constructed similarly as the N-pmCry2PHR-A1-mCherry-C plasmids except that primers for Gibson Assembly cloning were designed to only target the PrLD region of A1.

The N-GFP-G3BP1-C construct was generated from GFP (pcDNA3.1/NT-GFP-TOPO, Thermo Fisher Scientific, Burlington, ON, Canada) and G3BP1 (pDONR221-G3BP1, DNASU, Tempe, AZ, USA) cloned into the pmCry2PHR-mCherry (mCh) base vector (Plasmid 26866, Addgene, Watertown, MA, USA) between the NheI and MfeI restriction enzyme sites of the pmCry2PHR-mCh backbone, with the two-fragment Gibson Assembly. A short amino acid linker of GGSG (ggaggatcagga) was inserted during Gibson Assembly between GFP and G3BP1 to separate both proteins and prohibit unwanted non-specific interactions.

The expression of all constructs was driven by a CMV promoter. Sequencing of the constructs was performed to confirm correct orientation, and to confirm the absence of unwanted mutations. All primer sequences used for cloning are listed in Supplemental Table S1.

### 4.4. Blue Light Treatments

BL stimulation was performed in poly-D-lysine (Millipore-Sigma, Oakville, ON, Canada) coated 4-well glass-bottom slides (live-cell imaging), 8-well slides (fixed-cell imaging), or 6-well plates using custom-built LED arrays designed to fit multiple cell culture vessel dimensions and withstand common temperature/humidity requirements of cell culture incubators. LED arrays were positioned ~4.0 cm above the culture surface, and 10,000 lux (measurement of luminous energy per unit of time, per unit area) of 465nm BL was used to stimulate cultured cells.

### 4.5. Stress Treatments

Sodium arsenite (NaAsO_2_, 0.5 mM, 30 min) treatment was used as a positive control to induce SGs where indicated.

### 4.6. Nuclear/Cytoplasmic Fractionation and SDS-PAGE/Western Immunoblotting

Nuclear/cytoplasmic subcellular fractionation was performed using NE-PER Nuclear and Cytoplasmic Extraction Reagents Kit (Thermo Fisher Scientific, Burlington, ON, Canada) according to manufacturer′s instructions. Cytoplasmic protein concentrations for individual fractions were determined using A280 on a NanoDrop 1000 Spectrophotometer (Thermo Fisher Scientific, Burlington, ON, Canada) and subsequently analyzed by Western immunoblotting. Equivalent amounts of protein (40 µg) were separated by 10% SDS-PAGE and transferred to PVDF membranes (Millipore Sigma, Oakville, ON, Canada) using the Trans-Blot Semi-Dry Electrophoretic Transfer Cell System (Bio-Rad, Mississauga, ON, Canada); Ponceau S staining was used to assess the equivalency of protein loading after SDS-PAGE transfer. Membranes were washed with 1.0 M Tris-buffered saline (TBS), pH 7.4, blocked with 5% Bovine Serum Albumin (BSA) in TBS for 60 min at room temperature, and then incubated with primary antibodies (Table 1) in 5% BSA in TBS-T (0.1% Tween 20) overnight at 4 °C. Following TBS-T washes, membranes were incubated with peroxidase-conjugated secondary antibodies (Table 1) and protein bands were detected using Clarity Western ECL Substrate (Bio-Rad, Mississauga, ON, Canada) and visualized using the ChemiDoc Imaging System (Bio-Rad, Mississauga, ON, Canada).

### 4.7. Immunocytochemistry

Cells were prepared for immunocytochemistry (ICC) by initial fixation using 4.0% formaldehyde (prepared from a 37% stock; Millipore Sigma) in 1.0 M phosphate-buffer saline (PBS), pH 7.4, for 15 min at room temperature. After fixation, and three washes with PBS, cells were permeabilized with 0.3% Triton X-100 (Millipore Sigma, Mississauga, ON, Canada)/PBS for 30 min. Cells were washed three times with PBS and then blocked with Sea Block Blocking Buffer (Thermo Fisher Scientific) for 60 min at room temperature, blocking for non-specific antibody sites. Primary antibodies (Table 1) were diluted in 0.1% Triton X-100/PBS with 10% Sea Block and incubated overnight at 4 °C. Cells were then washed three times with PBS before incubation with secondary antibodies diluted in 0.1% Triton X-100/PBS with 5% Sea Block (Table 1) for 60 min at room temperature. Cells were then washed three times with PBS and mounted in ProLong Gold Antifade Mountant with DAPI (Fisher Scientific). All images and quantification are representative of three or more biological replicates. Random fields of view (RFV; ~5–6 per experiment) were acquired for quantification, and ~50–60 cells were analyzed per experiment. All images were acquired using identical settings within each experiment.

### 4.8. Live-Cell Imaging

Live-cell imaging was performed using an Axio Observer 7, inverted, fluorescent microscope (Carl Zeiss Canada Ltd., Toronto, ON, Canada), mounted with a P Lab-Tek^TM^ S1 heating insert (Carl Zeiss Canada Ltd., Toronto, ON, Canada) that was regulated by both a CO_2_ Module S1 and a TempModule S1 (Carl Zeiss Canada Ltd., Toronto, ON, Canada). Following transfections, medium was changed to FluoroBrite phenol red-free DMEM (Thermo Fisher Scientific, Burlington, ON, Canada), supplemented with 10% fetal bovine growth serum and 1% penicillin/streptomycin, and cells were equilibrated in the preheated heating insert (37 °C and 5% CO_2_) for 10 min prior to imaging. BL stimulation was performed using a custom-built blue light LED array that was positioned above the heating insert to stimulate cells in the live-cell incubation unit (~4.0 cm above the culture surface). Cells were stimulated with BL (465 nm, ~10,000 lux) for a period of 120 min to study A1 and G3BP1 punctum formation dynamics, immediately followed by a period of no BL for 60 min to study hnRNPA1 puncta dissociation dynamics, should puncta form. Live-cell images were captured using a Plan Apochromat 40× Oil objective, with a 1.40 numerical aperture, in one-minute intervals over the total 180 min. During the 120-min stimulation period, the BL LED was turned off for 10 s for imaging every 60 s, and the Zeiss ZEN 3.1 Blue microscope software captured mCherry signal in the 594 nm channel, and GFP in the 488 nm channel for G3BP1 experimentation. This was performed by synchronizing the LED and microscope using a Nearpow Multifunctional Infinite Cycle Programmable Plug-in Digital Timer Switch (Amazon, Seattle, WA, USA) attached to and controlling the LED array, and the Experimental Designer Module (Carl Zeiss Canada Ltd., Toronto, ON, Canada) in ZEN 3.1 Blue Edition (Carl Zeiss Canada Ltd., Toronto, ON, Canada) controlling both 594 nm and 488 nm stimulation and capture. Data presented are representative of at least three biological replicates, utilizing ~10 RFV per experiment. Overall, ~100 cells were analyzed per biological replicate.

### 4.9. Quantification and Statistical Analysis

#### 4.9.1. Immunocytochemistry

All image visualization and cell counting of transfected, non-cluster and cluster A1 containing cells were performed using ZEN 3.1 Blue Edition (Carl Zeiss Canada Ltd., Toronto, ON, Canada). Fixed-cell image quantification of mCherry A1 clusters and Alexa Fluor 488 nm G3BP1 puncta was examined using ImageJ, with Pearson’s correlation coefficient for A1 cluster and G3BP1 puncta co-localization calculated using the Coloc 2 plugin in ImageJ, with ~5 fields of view analyzed per condition, encompassing ~75 cells and ~200 clusters analyzed over triplicate experiments. Cytoplasmic/nuclear fluorescence intensity ratios were examined using ImageJ. Briefly, image scales were set for each image (taken consistently at 5 µm), and cellular nuclei and cytoplasm regions of interest (ROI) were hand drawn using DAPI (nuclei) and G3BP1 (cytoplasm) staining. The area (um^2^) and integrated density of mCherry was then measured for each ROI. In parallel, three background ROIs were taken and mean grey values were taken and averaged. The corrected total cellular fluorescence (CTCF) intensity was then analyzed using the formula: CTCF = Integrated Density − (Area of selected ROI * Average fluorescence of background readings). ~5 fields of view were analyzed per condition, encompassing ~50 cells analyzed per biological replicate.

#### 4.9.2. Live-Cell Imaging

Time-lapse image sequences acquired during high-throughput LED screening were visualized using ZEN 3.1 Blue Edition (Carl Zeiss Canada, Ltd., Toronto, ON, Canada). Analysis of cells containing A1 clusters was performed manually. Only cells that contained two distinctly observable A1 clusters at each time-point in data analyses were included in the final data analysis. The number of cells containing detectable A1 clusters were tracked over time and divided by the total number of cells within the imaging field to generate a percentage of cell with inclusions for each time-point within the imaging sequence.

Quantification of light-induced A1 cluster formation and dissociation was performed manually using ZEN 3.1 Blue Edition (Carl Zeiss Canada, Ltd., Toronto, ON, Canada), again analyzing only cells that contained two distinctly observable A1 clusters at each time-point in data analysis. For A1 cluster formation and dissociation rate quantification, cells with clusters were initially counted for each time-point. Each time-point cluster value was then converted to a percentage of the total cellular cluster maximum at the height of the stimulation cycle. Mean percentages for each time-point, from three biological replicates, were then plotted over-time for each stimulation cycle. For GFP-G3BP1 granule formation and dissociation, a similar analysis was performed.

Live-cell image quantification of A1 cluster amount and size was performed using ImageJ by first exporting live-cell images as individual 16-bit. TIFF images. A1 clusters were analyzed by thresholding individual cytoplasmic clusters and using the Analyze Particles tool in ImageJ. Four biological replicates were analyzed, encompassing ~200 puncta analyzed per replicate.

#### 4.9.3. Statistics

Statistical significance was calculated in GraphPad Prism software (Version 9.0.0) (GraphPad Software, San Diego, CA, USA) and resulting P values less than or equal to 0.05 were determined to be significant. One-way ANOVAs, with Tukey post hoc analysis, and two-way ANOVAs, with Bonferroni post hoc analysis, were used where indicated. To determine half-maximal formation and dissociation responses, graphs were subjected to a non-linear regression curve fit, using a dose–response, bi-phasic equation. *n* values described in the text and figure legends represent number of cells per experimental group, unless otherwise indicated, across multiple independent, biological experiments. Representative images were prepared using Adobe Photoshop CS6 (Adobe, San Jose, CA, USA).

## Figures and Tables

**Table 1:** Key resources table. Listed are the reagents/resources utilized in this study. Their purchase source and catalogue identifiers are included.

Reagent or Resource	Source	Identifier
**Antibodies**		
Mouse monoclonal anti-β-Actin (8H10D10)	New England BioLabs	Cat# 3700S
Rabbit monoclonal anti-EIF2S1 (EPR23098-50)	Abcam	Cat# ab242148
Rabbit monoclonal anti-EIF2S1 (phospho S51) (E90)	Abcam	Cat# ab32157
Rabbit polyclonal anti-G3BP (EPR13986(B))	Abcam	Cat# ab217225
Mouse monoclonal anti-hnRNPA1 (4B10)	Millipore Sigma	Cat# 05-1521
Rabbit polyclonal anti-TDP-43	Proteintech	Cat# 10782-2-AP
Alexa Fluor 488 Donkey anti-rabbit IgG (H + L)	Jackson ImmunoResearch	Cat# 711-546-152
Alexa Fluor 647 Goat anti-rabbit IgG (H + L)	Jackson ImmunoResearch	Cat# 111-605-047
Goat anti-mouse IgG (H + L) HRP Conjugate	Bio-Rad	Cat# 1706516
Goat anti-rabbit IgG (H + L) HRP Conjugate	Bio-Rad	Cat# 1706515
**Chemicals**		
Dulbecco′s Modified Eagle Media (DMEM)	Fisher Scientific	Cat# MT-10-013-CV
FluoroBrite DMEM Media	Fisher Scientific	Cat# A1896701
Formaldehyde, 37% by weight (with preservative/certified ACS)	Fisher Scientific	Cat# F79-500
Gibco Fetal Bovine Growth Serum	Thermo Fisher Scientific	Cat# 12483020
Invitrogen ProLong Gold Antifade Mountant with DAPI	Fisher Scientific	Cat# P36935
Immobilon-FL PVDF Membrane	Millipore Sigma	Cat# IPVH00010
Lipofectamine 2000	Fisher Scientific	Cat# 11-668-019
Opti-MEM Reduced Serum Media	Fisher Scientific	Cat# 31985062
Sodium Arsenite Solution	Millipore Sigma	Cat# 106277
Sodium Dodecyl Sulfate (SDS)	Fisher Scientific	Cat# BP166
Triton X-100	Fisher Scientific	Cat# AC215682500
Tween 20	Fisher Scientific	Cat# BP337
Commercial Assays		
Clarity Western ECL Substrate	Bio-Rad	Cat# 1705060
NE-PER Nuclear Cytoplasmic Extraction Reagents Kit	Thermo Fisher Scientific	Cat# 78833
**Cell Lines**		
HEK293T	ATCC	Cat# CRL-11268
**Recombinant DNA Plasmids**		
pmCry2PHR-mCherry	Addgene	Cat# 26866
pmTriEx-A1WT	[25]	N/A
pmTriEx-A1(F281L)	[25]	N/A
pmTriEx-A1(P275S)	[25]	N/A
pmA1WT-mCherry	This paper	N/A
pmA1(F281L)-mCherry	This paper	N/A
pmA1(P275S)-mCherry	This paper	N/A
pmCry2PHR-A1WT-mCherry	This paper	N/A
pmCry2PHR-A1(F281L)-mCherry	This paper	N/A
pmCry2PHR-A1(P275S)-mCherry	This paper	N/A
pmCry2PHR-A1(PrLD)-mCherry	This paper	N/A
pcDNA3.1/NT-GFP-TOPO	Thermo Fisher Scientific	Cat# K481001
pDONR221-G3BP1	DNASU	Cat# HsCD00042033
pmGFP-G3BP1	This paper	N/A
**Primers**		
Gibson Assembly Primers (See Supplemental Table S1)	IDT	N/A
**Software**		
Adobe Photoshop CS6	Adobe	https://www.adobe.com
GraphPad Prism 9.0.0	GraphPad Software Inc.	https://www.graphpad.com
ImageJ	NIH	https://imagej.nih.gov/ij/
ZEN 3.1 Blue Edition	Carl Zeiss Microscopy, LLC	https://www.zeiss.com

## Data Availability

Requests for data, resources and reagents should be directed to the co-corresponding author Michael C. Levin (michael.levin@usask.ca).

## Supplementary Materials

The following are available online at https://www.mdpi.com/1422-0067/22/6/2909/s1.

## References

[1] Lassmann H., van Horssen J. (2011). The molecular basis of neurodegeneration in multiple sclerosis. FEBS Lett..

[2] Dutta R., McDonough J., Yin X., Peterson J., Chang A., Torres T., Gudz T., Macklin W.B., Lewis D.A., Fox R.J. (2006). Mitochondrial dysfunction as a cause of axonal degenerationin multiple sclerosis patients. Ann. Neurol..

[3] De Vos K.J., Grierson A.J., Ackerley S., Miller C.C.J. (2008). Role of axonal transport in neurodegenerative diseases. Annu. Rev. Neurosci..

[4] Campbell G.R., Ziabreva I., Reeve A.K., Krishnan K.J., Reynolds R., Howell O., Lassmann H., Turnbull D.M., Mahad D.J. (2010). Mitochondrial DNA deletions and neurodegeneration in multiple sclerosis. Ann. Neurol..

[5] Haider L., Fischer M.T., Frischer J.M., Bauer J., Höftberger R., Botond G., Esterbauer H., Binder C.J., Witztum J.L., Lassmann H. (2011). Oxidative damage in multiple sclerosis lesions. Brain.

[6] Fischer M.T., Sharma R., Lim J.L., Haider L., Frischer J.M., Drexhage J., Mahad D., Bradl M., Van Horssen J., Lassmann H. (2012). NADPH oxidase expression in active multiple sclerosis lesions in relation to oxidative tissue damage and mitochondrial injury. Brain.

[7] Chen W.-W., Zhang X., Huang W.-J. (2016). Role of neuroinflammation in neurodegenerative diseases (Review). Mol. Med. Rep..

[8] Ransohoff R.M. (2016). How neuroinflammation contributes to neurodegeneration. Science.

[9] Douglas J.N., Gardner L.A., Levin M.C. (2013). Antibodies to an Intracellular Antigen Penetrate Neuronal Cells and Cause Deleterious Effects. J. Clin. Cell. Immunol..

[10] Kozin M.S., Kulakova O.G., Favorova O.O. (2018). Involvement of Mitochondria in Neurodegeneration in Multiple Sclerosis. Biochemstry.

[11] Trapp B.D., Stys P.K. (2009). Virtual hypoxia and chronic necrosis of demyelinated axons in multiple sclerosis. Lancet Neurol..

[12] Trapp B.D., Ransohoff R., Rudick R. (1999). Axonal pathology in multiple sclerosis: Relationship to neurologic disability. Curr. Opin. Neurol..

[13] Levin M.C., Lee S.M., Kalume F., Morcos Y., Dohan F.C., Hasty K.A., Callaway J.C., Zunt J., Desiderio D.M., Stuart J.M. (2002). Autoimmunity due to molecular mimicry as a cause of neurological disease. Nat. Med..

[14] De March A.K., De Bouwerie M., Kolopp-Sarda M.N., Faure G.C., Bene M.C., Bernard C.C.A. (2003). Anti-myelin oligodendrocyte glycoprotein B-cell responses in multiple sclerosis. J. Neuroimmunol..

[15] Craner M.J., Newcombe J., Black J.A., Hartle C., Cuzner M.L., Waxman S.G. (2004). Molecular changes in neurons in multiple sclerosis: Altered axonal expression of Nav1.2 and Nav1.6 sodium channels and Na^+^/Ca^2+^ exchanger. Proc. Natl. Acad. Sci. USA.

[16] Khalil M., Reindl M., Lutterotti A., Kuenz B., Ehling R., Gneiss C., Lackner P., Deisenhammer F., Berger T. (2006). Epitope specificity of serum antibodies directed against the extracellular domain of myelin oligodendrocyte glycoprotein: Influence of relapses and immunomodulatory treatments. J. Neuroimmunol..

[17] Black J.A., Newcombe J., Trapp B.D., Waxman S.G. (2007). Sodium Channel Expression Within Chronic Multiple Sclerosis Plaques. J. Neuropathol. Exp. Neurol..

[18] Dutta R., Trapp B.D. (2007). Pathogenesis of axonal and neuronal damage in multiple sclerosis. Neurological.

[19] Bar-Or A., Fawaz L., Fan B., Darlington P.J., Rieger A., Msc C.G., Calabresi P.A., Waubant E., Hauser S.L., Zhang J. (2009). Abnormal B-cell cytokine responses a trigger of T-cell-mediated disease in MS?. Ann. Neurol..

[20] Dutta R., Trapp B.D. (2011). Mechanisms of neuronal dysfunction and degeneration in multiple sclerosis. Prog. Neurobiol..

[21] Dendrou C.A., Fugger L., Friese M.A. (2015). Immunopathology of multiple sclerosis. Nat. Rev. Immunol..

[22] Li R., Patterson K.R., Bar-Or A. (2018). Reassessing B cell contributions in multiple sclerosis. Nat. Immunol..

[23] Lee S.M., Dunnavant F.D., Jang H., Zunt J., Levin M.C. (2006). Autoantibodies that recognize functional domains of hnRNPA1 implicate molecular mimicry in the pathogenesis of neurological disease. Neurosci. Lett..

[24] Levin M.C., Lee S., Gardner L.A., Shin Y., Douglas J.N., Cooper C. (2013). Autoantibodies to Non-myelin Antigens as Contributors to the Pathogenesis of Multiple Sclerosis. J. Clin. Cell Immunol..

[25] Lee S., Levin M. (2014). Novel somatic single nucleotide variants within the RNA binding protein hnRNP A1 in multiple sclerosis patients. F1000Research.

[26] Douglas J.N., Gardner L.A., Salapa H.E., Levin M.C. (2016). Antibodies to the RNA Binding Protein Heterogeneous Nuclear Ribonucleoprotein A1 Colocalize to Stress Granules Resulting in Altered RNA and Protein Levels in a Model of Neurodegeneration in Multiple Sclerosis. J. Clin. Cell. Immunol..

[27] Salapa H.E., Johnson C., Hutchinson C., Popescu B.F., Levin M.C. (2018). Dysfunctional RNA binding proteins and stress granules in multiple sclerosis. J. Neuroimmunol..

[28] Lee S., Salapa H.E., Levin M.C. (2019). Localization of near-infrared labeled antibodies to the central nervous system in experimental autoimmune encephalomyelitis. PLoS ONE.

[29] Libner C.D., Salapa H.E., Hutchinson C., Lee S., Levin M.C. (2019). Antibodies to the RNA binding protein heterogeneous nuclear ribonucleoprotein A1 contribute to neuronal cell loss in an animal model of multiple sclerosis. J. Comp. Neurol..

[30] Salapa H.E., Libner C.D., Levin M.C. (2019). Dysfunctional RNA-binding protein biology and neurodegeneration in experimental autoimmune encephalomyelitis in female mice. J. Neurosci. Res..

[31] Salapa H.E., Hutchinson C., Popescu B.F., Levin M.C. (2020). Neuronal RNA-binding protein dysfunction in multiple sclerosis cortex. Ann. Clin. Transl. Neurol..

[32] Singh R. (2002). RNA-protein interactions that regulate pre-mRNA splicing. Gene Expr..

[33] Kishore S., Luber S., Zavolan M. (2010). Deciphering the role of RNA-binding proteins in the post-transcriptional control of gene expression. Brief. Funct. Genom..

[34] Licatalosi D.D., Darnell R.B. (2010). RNA processing and its regulation: Global insights into biological networks. Nat. Rev. Genet..

[35] Han K., Yeo G., An P., Burge C.B., Grabowski P.J. (2005). A Combinatorial Code for Splicing Silencing: UAGG and GGGG Motifs. PLoS Biol..

[36] Roy R., Durie D., Lin G., Liu B.-Q., Skehel J.M., Mauri F., Cuorvo L.V., Barbareschi M., Guo L., Holcik M. (2014). hnRNPA1 couples nuclear export and translation of specific mRNAs downstream of FGF-2/S6K2 signalling. Nucleic Acids Res..

[37] Rebane A., Aab A., Steitz J.A. (2004). Transportins 1 and 2 are redundant nuclear import factors for hnRNP A1 and HuR. RNA.

[38] Allemand E., Guil S., Myers M., Moscat J., Cáceres J.F., Krainer A.R. (2005). Regulation of heterogenous nuclear ribonucleoprotein A1 transport by phosphorylation in cells stressed by osmotic shock. Proc. Natl. Acad. Sci. USA.

[39] He Y., Smith R. (2008). Nuclear functions of heterogeneous nuclear ribonucleoproteins A/B. Cell. Mol. Life Sci..

[40] Cartegni L., Maconi M., Morandi E., Cobianchi F., Riva S., Biamonti G. (1996). hnRNP A1 Selectively Interacts Through its Gly-rich Domain with Different RNA-binding Proteins. J. Mol. Biol..

[41] Fisette J.-F., Toutant J., Dugré-Brisson S., DesGroseillers L., Chabot B. (2009). hnRNP A1 and hnRNP H can collaborate to modulate 5’ splice site selection. RNA.

[42] Kim H.J., Kim N.C., Wang Y.-D., Scarborough E.A., Moore J., Diaz Z., MacLea K.S., Freibaum B., Li S., Molliex A. (2013). Mutations in prion-like domains in hnRNPA2B1 and hnRNPA1 cause multisystem proteinopathy and ALS. Nature.

[43] Molliex A., Temirov J., Lee J., Coughlin M., Kanagaraj A.P., Kim H.J., Mittag T., Taylor J.P. (2015). Phase Separation by Low Complexity Domains Promotes Stress Granule Assembly and Drives Pathological Fibrillization. Cell.

[44] Franzmann T.M., Alberti S. (2019). Prion-like low-complexity sequences: Key regulators of protein solubility and phase behavior. J. Biol. Chem..

[45] Lee B.J., Cansizoglu A.E., Suel K.E., Louis T.H., Zhang Z., Chook Y.M. (2006). Rules for nuclear localization sequence recognition by karyopherin beta 2. Cell.

[46] Lee S., Xu L., Shin Y., Gardner L., Hartzes A., Dohan F.C., Raine C., Homayouni R., Levin M.C. (2011). A potential link between autoimmunity and neurodegeneration in immune-mediated neurological disease. J. Neuroimmunol..

[47] De Conti L., Baralle M., Buratti E. (2017). Neurodegeneration and RNA-binding proteins. Wiley Interdiscip. Rev. RNA.

[48] Harrison A.F., Shorter J. (2017). RNA-binding proteins with prion-like domains in health and disease. Biochem. J..

[49] Guo L., Kim H.J., Wang H., Monaghan J., Freyermuth F., Sung J.C., O’Donovan K., Fare C.M., Diaz Z., Singh N. (2018). Nuclear-Import Receptors Reverse Aberrant Phase Transitions of RNA-Binding Proteins with Prion-like Domains. Cell.

[50] Mann J.R., Gleixner A.M., Mauna J.C., Gomes E., DeChellis-Marks M.R., Needham P.G., Copley K.E., Hurtle B., Portz B., Pyles N.J. (2019). RNA Binding Antagonizes Neurotoxic Phase Transitions of TDP-43. Neuron.

[51] Chung C.G., Lee H., Lee S.B. (2018). Mechanisms of protein toxicity in neurodegenerative diseases. Cell. Mol. Life Sci..

[52] Protter D.S., Rao B.S., Van Treeck B., Lin Y., Mizoue L., Rosen M.K., Parker R. (2018). Intrinsically Disordered Regions Can Contribute Promiscuous Interactions to RNP Granule Assembly. Cell Rep..

[53] Mittag T., Parker R. (2018). Multiple Modes of Protein–Protein Interactions Promote RNP Granule Assembly. J. Mol. Biol..

[54] Protter D.S., Parker R. (2016). Principles and Properties of Stress Granules. Trends Cell Biol..

[55] Wheeler J.R., Matheny T., Jain S., Abrisch R., Parker R. (2016). Distinct stages in stress granule assembly and disassembly. eLife.

[56] Hyman A.A., Weber C.A., Jülicher F. (2014). Liquid-Liquid Phase Separation in Biology. Annu. Rev. Cell Dev. Biol..

[57] Wang Q., Zuo Z., Wang X., Gu L., Yoshizumi T., Yang Z., Yang L., Liu Q., Liu W., Han Y.-J. (2016). Photoactivation and inactivation of Arabidopsis cryptochrome 2. Science.

[58] Zhang P., Fan B., Yang P., Temirov J., Messing J., Kim H.J., Taylor J.P. (2019). Chronic optogenetic induction of stress granules is cytotoxic and reveals the evolution of ALS-FTD pathology. eLife.

[59] Shin Y., Berry J., Pannucci N., Haataja M.P., Toettcher J.E., Brangwynne C.P. (2017). Spatiotemporal Control of Intracellular Phase Transitions Using Light-Activated optoDroplets. Cell.

[60] Tourriere H., Chebli K., Zekri L., Courselaud B., Blanchard J.M., Bertrand E., Tazi J. (2003). The RasGAP-associated endoribonuclease G3BP assembles stress granules. J. Cell Biol..

[61] Colombrita C., Zennaro E., Fallini C., Weber M., Sommacal A., Buratti E., Silani V., Ratti A. (2009). TDP-43 is recruited to stress granules in conditions of oxidative insult. J. Neurochem..

[62] Kedersha N., Chen S., Gilks N., Li W., Miller I.J., Stahl J., Anderson P. (2002). Evidence that ternary complex (eIF2-GTP-tRNA(i)(Met))-deficient preinitiation complexes are core constituents of mammalian stress granules. Mol. Biol. Cell.

[63] Takahashi M., Higuchi M., Matsuki H., Yoshita M., Ohsawa T., Oie M., Fujii M. (2013). Stress Granules Inhibit Apoptosis by Reducing Reactive Oxygen Species Production. Mol. Cell. Biol..

[64] Reineke L.C., Dougherty J.D., Pierre P., Lloyd R.E. (2012). Large G3BP-induced granules trigger eIF2α phosphorylation. Mol. Biol. Cell.

[65] Bracha D., Walls M.T., Wei M.-T., Zhu L., Kurian M., Avalos J.L., Toettcher J.E., Brangwynne C.P. (2018). Mapping Local and Global Liquid Phase Behavior in Living Cells Using Photo-Oligomerizable Seeds. Cell.

[66] Hutten S., Usluer S., Bourgeois B., Simonetti F., Odeh H.M., Fare C.M., Czuppa M., Hruska-Plochan M., Hofweber M., Polymenidou M. (2020). Nuclear Import Receptors Directly Bind to Arginine-Rich Dipeptide Repeat Proteins and Suppress Their Pathological Interactions. Cell Rep..

[67] Shorter J., Taylor J.P. (2013). Disease mutations in the prion-like domains of hnRNPA1 and hnRNPA2/B1 introduce potent steric zippers that drive excess RNP granule assembly. Rare Dis..

[68] Guil S., Long J.C., Cáceres J.F. (2006). hnRNP A1 Relocalization to the Stress Granules Reflects a Role in the Stress Response. Mol. Cell. Biol..

[69] Markmiller S., Soltanieh S., Server K.L., Mak R., Jin W., Fang M.Y., Luo E.-C., Krach F., Yang D., Sen A. (2018). Context-Dependent and Disease-Specific Diversity in Protein Interactions within Stress Granules. Cell.

[70] Youn J.-Y., Dyakov B.J., Zhang J., Knight J.D., Vernon R.M., Forman-Kay J.D., Gingras A.-C. (2019). Properties of Stress Granule and P-Body Proteomes. Mol. Cell.

[71] Gui X., Luo F., Li Y., Zhou H., Qin Z., Liu Z., Gu J., Xie M., Zhao K., Dai B. (2019). Structural basis for reversible amyloids of hnRNPA1 elucidates their role in stress granule assembly. Nat. Commun..

[72] Iervolino A., Santilli G., Trotta R., Guerzoni C., Cesi V., Bergamaschi A., Passerini C.G., Calabretta B., Perrotti D. (2002). hnRNP A1 Nucleocytoplasmic Shuttling Activity Is Required for Normal Myelopoiesis and BCR/ABL Leukemogenesis. Mol. Cell. Biol..

[73] Hamilton B.J., Burns C.M., Nichols R.C., Rigby W.F.C. (1997). Modulation of AUUUA Response Element Binding by Heterogeneous Nuclear Ribonucleoprotein A1 in Human T Lymphocytes. The roles of cytoplasmic location, transcription, and phosphorylation. J. Biol. Chem..

[74] Rajani D.K., Walch M., Martinvalet D., Thomas M.P., Lieberman J. (2012). Alterations in RNA processing during immune-mediated programmed cell death. Proc. Natl. Acad. Sci. USA.

[75] Allegretta M., Nicklas J.A., Sriram S., Albertini R.J. (1990). T cells responsive to myelin basic protein in patients with multiple sclerosis. Science.

[76] Lodge P.A., Allegretta M., Steinman L., Sriram S. (1994). Myelin basic protein peptide specificity and T-cell receptor gene usage of HPRT mutant T-cell clones in patients with multiple sclerosis. Ann. Neurol..

[77] Valori M., Jansson L., Kiviharju A., Ellonen P., Rajala H., Awad S.A., Mustjoki S., Tienari P.J. (2017). A novel class of somatic mutations in blood detected preferentially in CD8 + cells. Clin. Immunol..

[78] Van Horebeek L., Hilven K., Mallants K., Van Nieuwenhuijze A., Kelkka T., Savola P., Mustjoki S., Schlenner S.M., Liston A., Dubois B. (2019). A robust pipeline with high replication rate for detection of somatic variants in the adaptive immune system as a source of common genetic variation in autoimmune disease. Hum. Mol. Genet..

[79] Liu Q., Shu S., Wang R.R., Liu F., Cui B., Guo X.N., Lu C.X., Li X.G., Liu M.S., Peng B. (2016). Whole-exome sequencing identifies a missense mutation in hnRNPA1 in a family with flail arm ALS. Neurological.

[80] Naruse H., Ishiura H., Mitsui J., Date H., Takahashi Y., Matsukawa T., Tanaka M., Ishii A., Tamaoka A., Hokkoku K. (2018). Molecular epidemiological study of familial amyotrophic lateral sclerosis in Japanese population by whole-exome sequencing and identification of novel HNRNPA1 mutation. Neurobiol. Aging.

[81] Taylor J.P., Brown R.H., Cleveland D.W. (2016). Decoding ALS: From genes to mechanism. Nat. Cell Biol..

[82] Deeb O., Nabulsi M. (2020). Exploring Multiple Sclerosis (MS) and Amyotrophic Lateral Scler osis (ALS) as Neurodegenerative Diseases and their Treatments: A Review Study. Curr. Top. Med. Chem..

[83] Bahi-Buisson N., Souville I., Fourniol F.J., Toussaint A., Moores C.A., Houdusse A., Lemaitre J.Y., Poirier K., Khalaf-Nazzal R., Hully M. (2013). New insights into genotype–phenotype correlations for the doublecortin-related lissencephaly spectrum. Brain.

[84] Gomez-Ramos A., Picher A.J., García E., Garrido P., Hernandez F., Soriano E., Avila J. (2017). Validation of Suspected Somatic Single Nucleotide Variations in the Brain of Alzheimer’s Disease Patients. J. Alzheimer’s Dis..

[85] McConnell M.J., Moran J.V., Abyzov A., Akbarian S., Bae T., Cortes-Ciriano I., Erwin J.A., Fasching L., Flasch D.A., Freed D. (2017). Intersection of diverse neuronal genomes and neuropsychiatric disease: The Brain Somatic Mosaicism Network. Science.

