# Supplementary material for: Multiple Sclerosis-Associated hnRNPA1 Mutations Alter hnRNPA1 Dynamics and Influence Stress Granule Formation
Source: Int J Mol Sci. 2021 Mar 12;22(6):2909. doi: 10.3390/ijms22062909 (PMC7998649; doi:10.3390/ijms22062909)
Supplement: Supplementary file 1 [file ijms-22-02909-s001.zip › ijms-1127258 suppl/Table S1.docx]

**Supplemental Table 1: Gibson Assembly Cloning Primers and PCR Products**

| **DNA Input** | **Forward Primer** | **Reverse Primer** | **PCR Product** |
| --- | --- | --- | --- |
| pmTriEx-A1WT | TTA GTG AAC CGT CAG ATC CGC TAG CAT GTC TAA GTC AGA GTC TCC | TCC TGA TCC TCC AAA TCT TCT GCC ACT GCC | A1WT |
| pmTriEx-A1(F281L) | TTA GTG AAC CGT CAG ATC CGC TAG CAT GTC TAA GTC AGA GTC TCC | TCC TGA TCC TCC AAA TCT TCT GCC ACT GCC | F281L |
| pmTriEx-A1(P275S) | TTA GTG AAC CGT CAG ATC CGC TAG CAT GTC TAA GTC AGA GTC TCC | TCC TGA TCC TCC AAA TCT TCT GCC ACT GCC | P275S |
| pmCry2PHR-mCherry | CAG AAG ATT TGG AGG ATC AGG AAT GGT GAG CAA GGG CGA G | ATA AAC AAG TTA ACA ACA ACC TAC TTG TAC AGC TCG TCC ATG | mCherry |
| pmCry2PHR-mCherry | TTA GTG AAC CGT CAG ATC CGA TGA AGA TGG ACA AAA AGA CTA TAG TTT GG | GAG ACT CTG ACT TAG ACA TGC CTG ATC CTC CGG CTG CTG CTC CGA TCA TG | Cry2PHR |
| pcDNA3.1/NT-GFP-TOPO | CTG GTT TAG TGA ACC GTC AGA TCC GAT GGC CAG CAA AGG AGA AG | TCC TGA TCC TCC ATC CAT GCC ATG TGT AAT CC | GFP |
| pmGFP-G3BP1 | ACA TGG CAT GGA TGG AGG ATC AGG AAT GGT GAT GGA GAA GCC TAG TCC CC | CTG CAA TAA ACA AGT TAA CAA CAA CTT ACT GCC GTG GCG CAA GCC C | G3BP1 |
