# Supplementary figures and images for: Multiple Sclerosis-Associated hnRNPA1 Mutations Alter hnRNPA1 Dynamics and Influence Stress Granule Formation
Source: Int J Mol Sci. 2021 Mar 12;22(6):2909. doi: 10.3390/ijms22062909 (PMC7998649; doi:10.3390/ijms22062909)

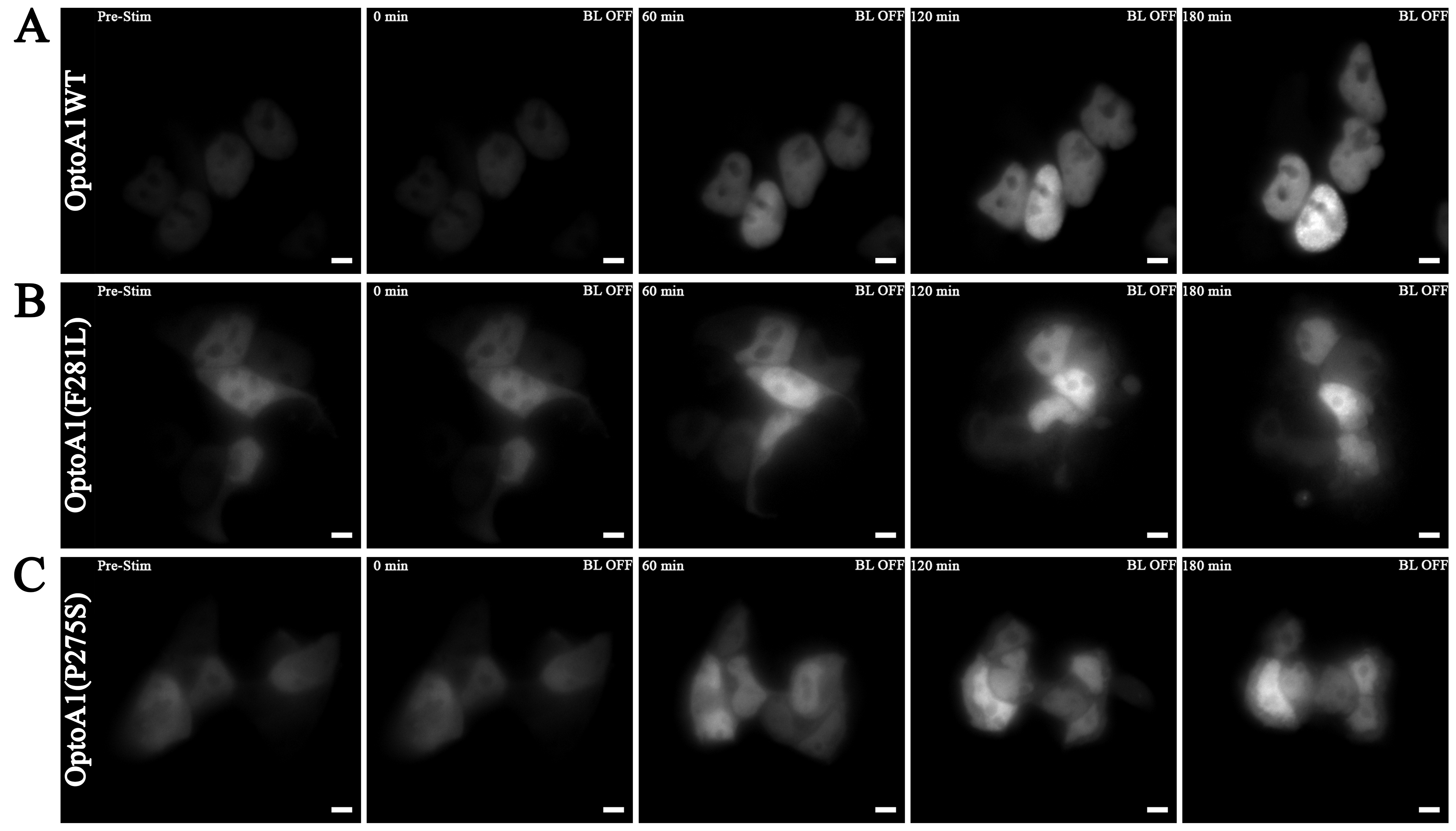

Supplement: Supplementary file 1 [file ijms-22-02909-s001.zip › ijms-1127258 suppl/Figure S1.tif]

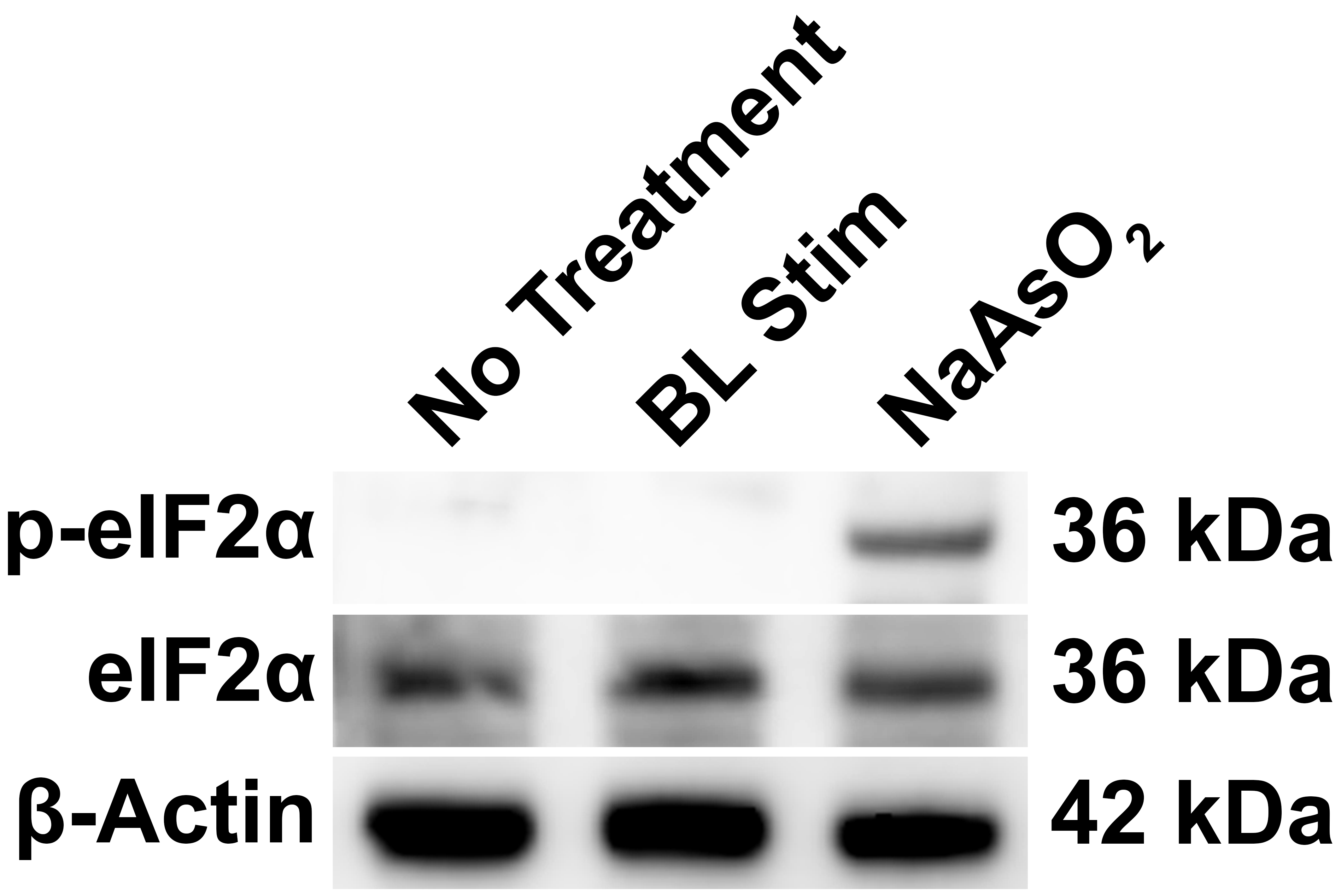

Supplement: Supplementary file 1 [file ijms-22-02909-s001.zip › ijms-1127258 suppl/Figure S2.tif]

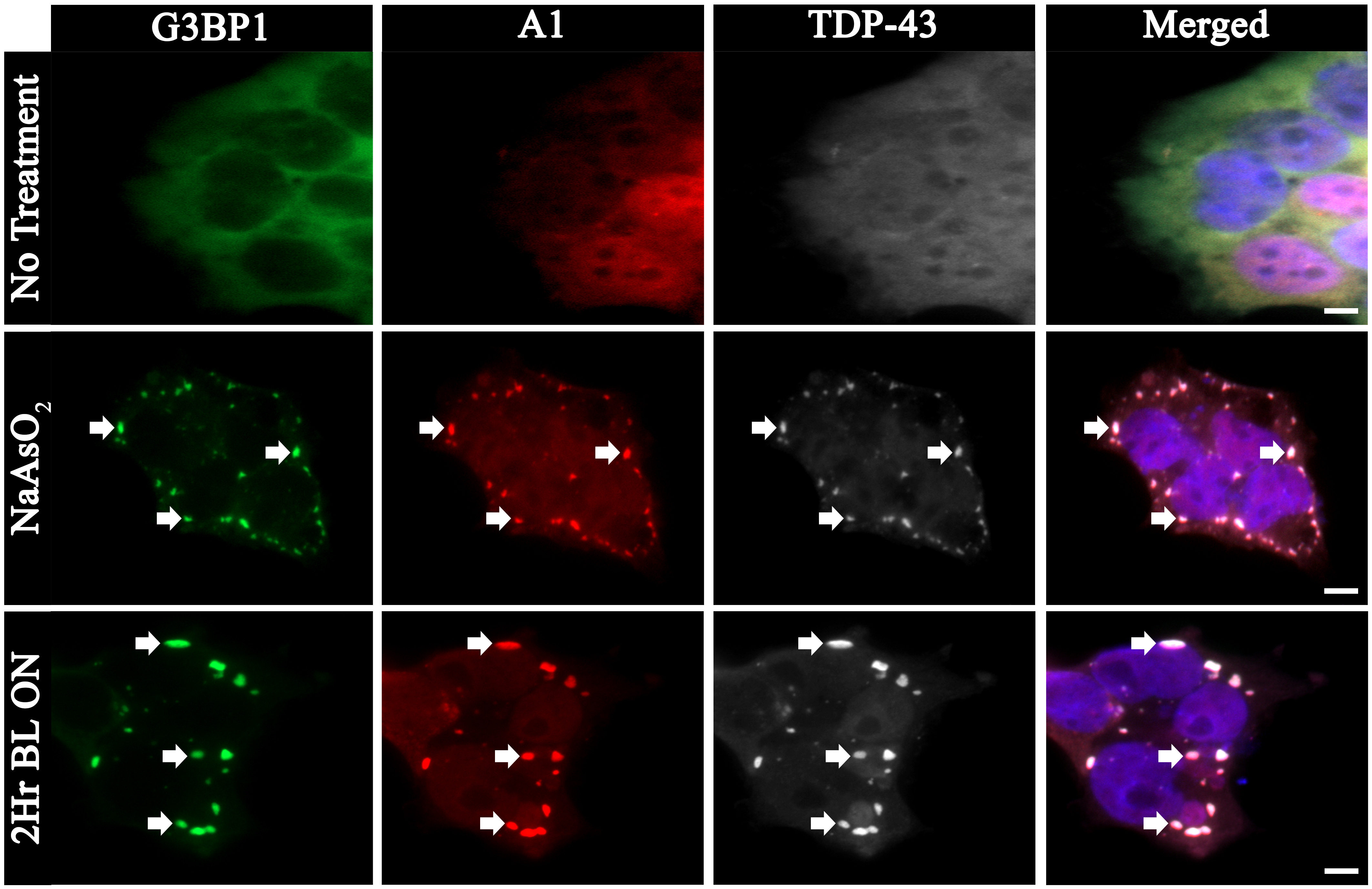

Supplement: Supplementary file 1 [file ijms-22-02909-s001.zip › ijms-1127258 suppl/Figure S3.tif]
